# Incidence and Risk Factors for Postoperative Complications in Patients Undergoing Extraoral Drainage for Maxillofacial Abscess: A Retrospective Cohort Study

**DOI:** 10.3390/jcm14103368

**Published:** 2025-05-12

**Authors:** Gregoire Longchamp, Harald Essig, Valerian Dirr, Marc M. Precht, Maximilian E. H. Wagner, Raphael Ferrari

**Affiliations:** Department of Cranio-Maxillofacial and Oral Surgery, University Hospital of Zurich, 8091 Zurich, Switzerland; harald.essig@usz.ch (H.E.); valerian.dirr@usz.ch (V.D.); marcmichael.precht@usz.ch (M.M.P.); maximilian.wagner@usz.ch (M.E.H.W.); raphael.ferrari@usz.ch (R.F.)

**Keywords:** abscess, drainage, infections, morbidity, postoperative complications, risk factors

## Abstract

**Background:** The standard treatment for maxillofacial abscesses is surgical drainage combined with antibiotics, a frequent procedure in maxillofacial surgery departments. However, postoperative complications following this surgery are poorly described in the literature. Identifying their incidence and risk factors could help improve patient outcomes and healthcare planning. **Objectives:** The primary aim was to identify postoperative complications within 30 days after maxillofacial abscess surgery; the secondary aim was to explore their associated risk factors. **Methods:** A monocentric retrospective cohort study included patients with maxillofacial abscesses who underwent extraoral incision and drainage under general anesthesia at the Department of Cranio-Maxillofacial and Oral Surgery at a tertiary hospital in Switzerland between January 2012 and August 2023. Postoperative complications within 30 days were recorded and classified according to the validated Dindo–Clavien classification system. Univariable and multivariable logistic regression analyses were conducted to identify risk factors for postoperative complications. **Results:** A total of 253 participants were analyzed. The overall complication rate was 24.1%, with 15.8% major complications (Dindo–Clavien grade ≥ 3). The most common minor complications (Dindo–Clavien grade < 3) were hypokalemia and lower-extremity edema, with an incidence of 6.4%. The most common major complications were persistent and recurrent abscesses, with an incidence rate of 10.4%. These complications occurred in the early (median range 1–5 days) and delayed (median range 14–15 days) postoperative courses, respectively. Overall, the mortality rate was 0.4%. On multivariable analysis, an American Society of Anesthesiologists (ASA) score > 2 was associated with overall and major postoperative complications (odds ratio [OR], 3.38; 95% CI 1.75–6.51; *p* < 0.001 and OR, 3.76; 95% CI 1.83–7.72; *p* < 0.001, respectively). Additionally, female sex (OR, 1.97; 95% CI 1.05–3.70; *p* = 0.036) and C-reactive protein level > 50 mg/L (OR, 2.25; 95% CI 1.01–4.98; *p* = 0.046) were associated with overall postoperative complications. **Conclusions:** This study introduces a novel application of the Dindo–Clavien classification to maxillofacial abscess surgery, providing a standardized framework for assessing postoperative complication severity. Through this approach, we identified ASA score > 2, female sex, and CRP level > 50 mg/L as risk factors for postoperative complications. Our findings highlight the importance of close monitoring during the first five postoperative days to detect early complications, such as persistent abscesses, and recommend standardized outpatient follow-up for at least two weeks to identify delayed complications, like recurrence.

## 1. Introduction

Maxillofacial infection is a common cause of admission to cranio-maxillofacial surgery departments [1]. A nationwide database from 2005 to 2022 in Germany reported an incidence rate of 9.8 per 100,000 person-years, with an increasing trend over time [2]. Prompt treatment is essential to prevent the spread of the infection and severe complications, such as airway obstruction, septic shock, or cavernous sinus thrombosis [3]. Surgical incision with drainage, combined with antibiotic therapy, remains the gold standard of treatment [4]. While initial-stage infections may be drained intraorally under local anesthesia, extensive collections require extraoral drainage under general anesthesia [5].

While the management of maxillofacial abscesses is well established, the postoperative course and potential complications have received less attention. Postoperative complications, defined as unexpected deviation from the typical recovery process after surgery, should be taken into account when performing inpatient procedures. Complications can be classified using the Dindo–Clavien (DC) classification system [6], which includes five grades: grade 1: no therapy required (allowed therapies included antiemetics, antipyretics, analgesics, diuretics, electrolytes, and physiotherapy); grade 2: requiring medical management; grade 3: requiring intervention; grade 4: life-threatening; and grade 5: mortality. Grades 3 and 4 are further subdivided by severity, grade 3a: no general anesthesia required; grade 3b: general anesthesia required; grade 4a: single-organ dysfunction; and grade 4b: multiorgan dysfunction.

Postoperative complications may lead to an increased length of stay, higher costs, and decreased quality of life [7]. However, postoperative complications after maxillofacial abscess surgery have been inconsistently described in the literature, and their predictors are controversial. In their retrospective study, Pham Dang et al. [8] analyzed 653 patients treated with drainage for maxillofacial infections. This study showed an increased risk of reoperation in the presence of penicillin allergy, psychiatric disorders, oropharyngeal edema, floor edema, and trismus upon admission. In another study [9], the surgical reintervention rate was significantly higher in patients with diabetes mellitus. In a study by Mathew et al. [10], multiple space involvement and a white blood cell count > 15 G/L were independent predictors of life-threatening complications. Despite these findings, the specific incidence, nature, and predictive factors of postoperative complications after maxillofacial abscess surgery remain unclear. Moreover, previous studies on complications after maxillofacial abscess surgery frequently exhibit key limitations, including heterogeneous patient populations, such as patients drained under local or general anesthesia. Additionally, these studies often use inconsistent or non-standardized definitions of complications.

Identifying postoperative complications and understanding their risk factors can help clinicians identify high-risk patients early, tailor postoperative surveillance, and plan appropriate follow-up strategies to reduce complication-related morbidity. Therefore, this study aimed to identify postoperative complications within 30 days after maxillofacial abscess surgery using the validated DC classification system. The secondary aim was to determine risk factors associated with these complications. We hypothesized that selected pre- and perioperative variables would be able to predict complications.

## 2. Materials and Methods

### 2.1. Study Design and Study Population

A retrospective analysis was conducted at the Department of Cranio-Maxillofacial and Oral Surgery at a tertiary hospital in Switzerland. The period spanned from 1 January 2012 to 31 August 2023. We included patients aged 18 years or older with a diagnosis of an odontogenic maxillofacial abscess who underwent extraoral incision and drainage. The indications for imaging and surgical intervention followed previously described criteria [11]. We excluded patients under 18 years, patients with surgical site infections (as defined by the Centers for Disease Control and Prevention [12]), and patients with peritonsillar abscesses or para-retropharyngeal abscesses.

The study was conducted in accordance with the Declaration of Helsinki and was approved by the local ethics committee (2023-01857). The requirement for informed consent was waived by the Institutional Review Board, given the retrospective design of the study. The study methods adhered to the Strengthening the Reporting of Observational Studies in Epidemiology (STROBE) guidelines and checklist (Table S1) [13].

### 2.2. Surgical Intervention and Perioperative Management

All extraoral surgical procedures were conducted under general anesthesia, and the surgical team always included at least one specialist from the Cranio-Maxillofacial and Oral Surgery department. The choice of surgical approach was determined based on the abscess localization. For the cervical approach, a skin incision was made two finger widths below the lower border of the mandible, followed by blunt subplatysmal dissection using curved pean forceps up to the mandibular bone. The infected anatomical space was opened by blunt dissection along the mandible towards the lingual and vestibular borders. If discharge was observed, microbiological sampling was performed. The decision to remove the tooth for source control was based on the clinical situation, risk of osteomyelitis or osteoradionecrosis (e.g., presence of antiresorptive therapy, head and neck radiotherapy, immunosuppression, or radiological signs of osteomyelitis on preoperative imaging), need for alveotomy, and likelihood of preserving the tooth. Surgical drains were placed, and the cavity was irrigated with either 0.9% sodium chloride solution or lactated Ringer’s solution. Drains remained in place postoperatively and were removed once clinical signs indicated resolution of the abscess, including decreased swelling, increased mouth opening, absence of pus, and clear rinsing fluid. Following surgery, all patients stayed in the hospital for close observation, intravenous antibiotic therapy, and daily irrigation of the abscess cavity through the surgical drains. Antibiotics were administered intravenously during hospital stay (amoxicillin–clavulanate 2.2 g every 8 h or clindamycin 600 mg every 8 h in case of penicillin allergy), and switched to the oral route after discharge (amoxicillin–clavulanate 1 g every 12 h or clindamycin 600 mg every 8 h in case of penicillin allergy). If necessary, antibiotic therapy was adjusted based on the results of intraoperative microbiological cultures. The total duration of antibiotic treatment was set to a minimum of 5 days, with adjustments made according to surgical observations, the patient’s condition, and changes in laboratory markers.

### 2.3. Outcome Measures

The primary outcome was the incidence of postoperative complications within 30 days, defined as any unexpected or adverse postoperative course requiring intervention. Complications were classified using the validated DC classification system [6]. Complications with a DC grade of ≥3 were defined as major, while DC grade < 3 were defined as minor. Postoperative complications were recorded throughout the inpatient treatment period and during the outpatient follow-up visits for up to 30 days postoperatively. A recurrent abscess was defined as the clinical and/or radiological reappearance of the abscess after initial recovery, whereas a persistent abscess was defined as one that showed no signs of recovery. The secondary outcomes included identification of predictive factors for overall and major postoperative complications. Predictors were selected a priori based on their clinical relevance and potential associations with postoperative complications based on previous studies [8,9,10]. Data included demographics, comorbidities, laboratory values, operative details, and perioperative outcomes. A detailed list of variables is provided in Supplementary Materials (File S1).

### 2.4. Statistical Analysis

Descriptive statistics were applied to analyze the data. Continuous variables were reported as mean ± standard deviation (SD) or median with range, depending on the distribution of the data. Discrete variables were presented as the number of patients (*n*) and their corresponding percentages. The chi-square test or Fisher’s exact test was used to detect differences in categorical variables, and an independent t-test was used for continuous variables. All the assumptions were met for normality. Univariable and multivariable logistic regression analyses were performed to identify the association between predictors and the occurrence of complications, reporting their odds ratios (OR), and their 95% confidence intervals (CI). Initially, univariable logistic regression was performed to evaluate the relationship between outcomes and predictors. Variables with *p* < 0.2 from the univariate analysis were subsequently included in a backward stepwise logistic regression to determine independent predictors of complications. To prevent collinearity between the ASA score and comorbidities (immunosuppression, chronic renal failure, diabetes mellitus, hypertension, cardiac insufficiency, or chronic respiratory insufficiency), only the ASA score was entered in the multivariable regression model. Statistical significance was set at *p* < 0.05. The goodness-of-fit of the final multivariable model was evaluated using the Hosmer–Lemeshow test. No imputation was performed for missing data. All statistical analyses were performed using STATA version 16.1 (Stata Corp., College Station, TX, USA, 2007).

## 3. Results

### 3.1. Baseline Characteristics

**Figure 1:**
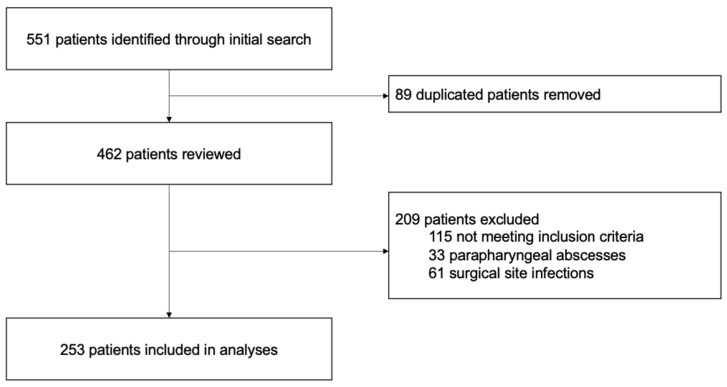
Study flowchart.

A total of 253 patients with maxillofacial abscesses who underwent extraoral incision and drainage under general anesthesia met the inclusion criteria (Figure 1). Of these, 107 (42.3%) were women and 59 (23.3%) were aged > 60 years at admission (Table S2). The most common comorbidity was hypertension (52 [20.6%]), and 57 (22.5%) had an American Society of Anesthesiologists (ASA) score > 2. Most patients had an increased white blood cell count > 10 G/L (180 [71.2%]) and C-reactive protein (CRP) level > 50 mg/L (184 [72.7%]), whereas only 33 patients (13.0%) had fever. Moreover, the mean white blood cell count and CRP level were 12.7 G/L and 115 mg/L, respectively. Overall, 181 patients (71.5%) underwent surgery within 24 h, and the operation duration was less than 30 min in 132 patients (52.2%) (Table S3). A cervical approach was performed in 223 cases (88.1%), with an additional intraoral approach in 110 cases (43.5%). Overall, 125 patients (49.4%) required an additional surgical procedure, with tooth extraction being the most common (94 [37.2%]). This was followed by intraoral incision and drainage (23 [9.1%]); other procedures such as partial mandibulectomy, sequestrectomy, or biopsy (6 [2.4%]); and tracheotomy (2 [0.8%]). The mean duration of hospitalization was 6.6 ± 4.0 days (Table S2).

### 3.2. Rate of Complications

**Figure 2:**
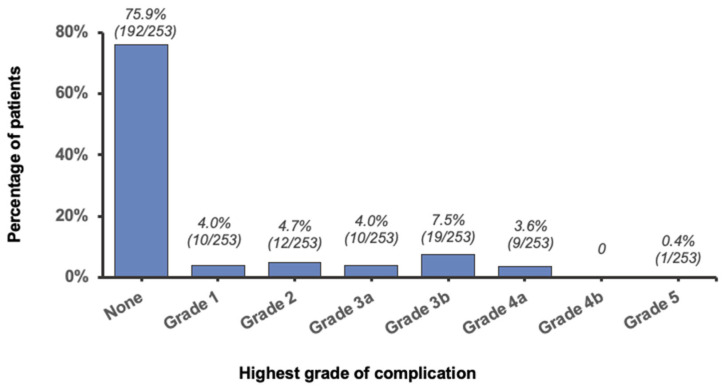
Distribution of the highest grade of complication.

In the full cohort, postoperative complications occurred within 30 days in 61 patients (24.1%; Figure 2). The most severe complication observed was grade 3b in 19 patients (7.5%), followed by grade 2 in 12 (4.7%), and grade 1 or grade 3a in 10 (4.0%). Only one patient died (grade 5, 0.4%), and no patient experienced a grade 4b complication.

Minor complications (DC grade < 3) are presented in Table 1. Overall, 25 (9.9%) DC grade 1 and 18 (7.1%) DC grade 2 complications were observed, and consisted mostly of medical complications. These complications occurred after a median of 5 days (range 1–16) and 3 days (range 0–20), respectively. The most common minor complications were hypokalemia and lower-extremity edema (each 8 cases [3.2%]), which required electrolytes and diuretics, respectively. The therapies for postoperative complications are shown in Table S4. One patient presented with progressive neck swelling 8 days after surgery. A CT scan revealed soft tissue thickening without fluid collection, and the patient was readmitted for intravenous antibiotics. Another 74-year-old patient experienced a non-ST segment elevation myocardial infarction on the second postoperative day but declined catheterization and was treated conservatively.

Moreover, 40 major complications (15.8%, DC grade ≥ 3) were observed (Table 1). The most common major complication was a persistent abscess requiring drainage under local (3 [1.2%]) or general anesthesia (12 [4.8%]). Of these, two (0.8%) were transferred to the intensive care unit postoperatively due to airway compromise. Other reasons for reoperation under general anesthesia included recurrent abscess (8 [3.2%]) and bleeding from the incision (1 [0.4%], temporal incision). Another patient was hospitalized with moderate perimandibular swelling without dyspnea. Due to the presence of an intraoral swelling, an initial intraoral drainage was performed, followed by an extraoral incision 26 h later. Due to airway compromise identified during intubation, the patient was transferred to the intensive care unit until the first postoperative day. Persistent abscesses were diagnosed earlier than recurrent abscesses (median range 1–5 days versus 14–15 days, respectively). Other major complications were diagnosed during the early postoperative period (range 0–10 days). In the entire cohort, only one patient died postoperatively (DC grade 5). This was an 87-year-old woman with an odontogenic perimandibular abscess and an ASA score 4, who presented with a deteriorated general condition, severe neck swelling, dyspnea, and stridor. She underwent emergency tracheotomy, abscess drainage, and removal of the affected teeth. The patient required reoperation due to abscess progression into the mediastinum but died of a multi-organ failure on postoperative day 4 due to septic shock, with the abscess being a contributing factor alongside significant comorbidities.

### 3.3. Predictive Factors

The preoperative and perioperative predictors associated with complications in the univariable analysis are described in Table 2 and Table 3, respectively. Preoperative factors associated with overall complications included age > 60 years (OR, 2.62 [95%CI, 1.39–4.93]; *p* = 0.003), female sex (OR, 1.88 [95%CI, 1.05–3.36]; *p* = 0.033), ASA score > 2 (OR, 3.76 [95%CI, 1.99–7.11]; *p* < 0.001), immunosuppression (OR, 4.02 [95%CI, 1.30–12.46]; *p* = 0.016), chronic renal failure (OR, 4.85 [95%CI, 1.48–15.89]; *p* = 0.009), cardiac insufficiency (OR, 4.68 [95%CI, 1.56–14.08]; *p* = 0.006), and mandibular incisor or canine as implicated tooth (OR, 0.29 [95%CI, 0.10–0.86]; *p* = 0.026). None of the perioperative factors were significantly associated with overall complications.

Factors that were associated with major complications (DC grade ≥ 3) in univariable analysis included ASA > 2 (OR, 3.76 [95%CI, 1.83–7.72]; *p* < 0.001), cardiac insufficiency (OR, 4.68 [95%CI, 1.53–14.36]; *p* = 0.007), and intraoral drainage as additional surgical procedure (OR, 3.27 [95%CI, 1.16–9.23]; *p* = 0.026).

In multivariable analysis (Table 4), three independent predictors of overall postoperative complications were identified as follows: female sex (OR, 1.97 [95%CI, 1.05–3.70]; *p* = 0.036), ASA score > 2 (OR, 3.38 [95%CI, 1.75–6.51]; *p* < 0.001), and CRP level > 50 mg/L (OR, 2.25 [95%CI, 1.01–4.98]; *p* = 0.046). For the prediction of major postoperative complications, only ASA score > 2 remained significant after backward stepwise logistic regression (OR, 3.76 [95%CI, 1.83–7.72]; *p* < 0.001). The Hosmer–Lemeshow goodness-of-fit test indicated adequate model calibration (*p* = 0.60).

## 4. Discussion

This study systematically evaluated postoperative complications within 30 days following incision and drainage for maxillofacial abscesses under general anesthesia, using an established classification system to stratify complications based on severity, ensuring a structured and reproducible assessment. The overall rate of postoperative complications was 24.1%, of which 15.8% were major complications. Hypokalemia and lower-extremity edema were the most frequent minor complications, with an incidence rate of 6.4%. Furthermore, persistent and recurrent abscesses were the most common major complications with incidence rates of 6.0% and 4.4%, respectively. These complications arose at different postoperative phases: persistent abscesses manifested early (median range 1–5 days), whereas recurrent abscesses developed later (median range 14–15 days). These findings support the importance of inpatient surveillance during the first postoperative days, as well as postoperative follow-up two weeks after surgery. Additional follow-up visits in between should be based on clinical judgement. Such follow-up facilitates the early detection of complications and is particularly crucial for patients with risk factors. In multivariable analysis, ASA score was a strong predictor of both overall and major postoperative complications. Additionally, female sex and CRP level > 50 mg/L were independent predictors of overall postoperative complications.

Previous studies reported similar postoperative complication rates following maxillofacial abscess surgery, ranging from 15% to 24% [10,14]. The variation in these rates can be attributed to differences in inclusion criteria, definitions of complications, and follow-up durations across studies. In our study, we also included minor complications as defined by the DC classification, when they required intervention. This approach allowed us to capture the full spectrum of postoperative care needs, which may explain why our complication rate of 24.1% is at the higher end of the reported range. Future research should explore whether alternative antibiotic regimens, such as combination therapy with metronidazole or extended treatment durations, could reduce complication rates.

Moreover, the reoperation rate under general anesthesia due to recurrence or persistence was 7.9% in the present study, consistent with previous studies reporting surgical reintervention rates ranging from 6 to 8% [8,9]. The perioperative mortality rate of 0.4% observed in this study was lower than the 0.7–11.3% reported in previous studies [15]. Other complications documented in prior research included transfer to the intensive care unit, tracheotomy, renal failure, and pleural effusion [9,10,14]. However, a detailed description of postoperative complications following maxillofacial abscess surgery remains scarce in the literature. To the best of our knowledge, no grading system has previously been applied to report these complications. In this study, we used the DC classification because it is easily applicable and demonstrated good interrater reliability [6]. This makes it an effective tool for standardizing the reporting of postoperative complications [16], and has already been used in head and neck surgeries [17]. Implementing a standardized grading system could enhance the consistency and clarity of reporting, and ultimately improve patient care following maxillofacial abscess surgery.

The present study also investigated the association between pre- and perioperative characteristics and postoperative complications. Previous studies have reported that comorbidities, such as psychiatric disorder [8], diabetes [9,10,14,18], chronic renal failure [14], and hypertension [18], were associated with complications in patients with maxillofacial abscesses. In the present study, diabetes mellitus was not significantly associated with postoperative complications, possibly due to effective preoperative management, differences in disease severity among patients, or variations in the definition and classification of complications compared to other studies. On the other hand, the presence of immunosuppression, chronic renal failure, or cardiac insufficiency was associated with overall postoperative complications in univariable logistic regression analysis. Additionally, the ASA score was a strong predictor in both univariable and multivariable analyses. Patients with an ASA score > 2 were 3.38 times more likely to experience overall complications (*p* < 0.001) and 3.76 times more likely to experience major complications (*p* < 0.001), when compared to patients with an ASA score ≤ 2. This score has some limitations owing to its subjective components, which impact its reliability and reproducibility [19]; nevertheless, it remains a widely used and simple score. Although the association between ASA score and postoperative complications after maxillofacial abscess surgery has not been investigated, a high ASA score was associated with an increased risk of complications after facial fracture repair [20], and other surgical procedures [19,21].

Another predictor of postoperative complications in the present study was an increased CRP level at admission > 50 mg/L (OR, 2.25; *p* = 0.046), consistent with the findings by Pham Dang et al. [8]. The latter study retrospectively analyzed 653 patients diagnosed with odontogenic infections who underwent drainage using an intraoral and/or transcervical approach. Their findings indicated that a CRP level of >200 mg/L was significantly associated with the need for multiple surgeries (OR, 4.12; *p* = 0.01). The discrepancy in CRP threshold is likely due to the different outcomes measured: our study encompassed a wide range of complications, including less severe events, while Pham Dang et al. targeted severe cases requiring multiple surgeries. Consequently, our lower threshold is more sensitive for detecting a broader spectrum of postoperative complications, whereas their higher threshold is more specific for identifying patients with advanced disease necessitating multiple interventions. However, as inflammatory responses evolve, serial CRP measurements may offer superior predictive accuracy. For instance, in oral oncologic surgery, postoperative serial CRP monitoring was highly sensitive for detecting early infectious complications [22]. Similarly, a meta-analysis on abdominal surgery found that serial CRP measurements effectively ruled out infectious complications with high negative predictive values [23]. Thus, future studies incorporating serial CRP assessments could enhance the prediction of complications following maxillofacial abscess surgery.

Another inflammatory parameter associated with complications in odontogenic infections was a white blood cell count > 15 G/L (OR, 3.84; *p* = 0.01) [10]. Additionally, multiple space involvement was another independent predictor of complications, with an odds ratio of 13.22; however, the wide confidence interval (1.7 to 102.1; *p* < 0.001) suggests variability in the estimate. Increased inflammatory parameters and multiple space involvement have also been shown in previous studies to be associated with increased length of stay or disease severity [11,24]. Given the heightened risk of complications in these patients, close monitoring from hospital admission to postoperative follow-up is essential. In cases of inadequate clinical improvement, a lower threshold for performing a CT scan to identify possible undrained collections should be considered.

Moreover, female sex was identified as an independent predictor of postoperative complications, with higher odds of complications than male sex (OR, 1.97; *p* = 0.036). In contrast, two other studies found no significant association between sex and complications [8,10]. Female sex as a predictor may reflect biological differences or confounders such as delayed presentation. Larger multicenter cohorts are needed to clarify whether this factor plays a significant role in predicting complications following maxillofacial abscess surgery.

Notably, only patient-specific factors were identified as independent predictors of postoperative complications. None of the perioperative variables, such as time to operation, operation duration, or number of drains, were significantly associated with complications in the multivariable model. In the univariable analysis, intraoral incision and drainage in addition to extraoral drainage was the only perioperative factor associated with major complications (OR, 3.27; *p* = 0.026). This association may reflect underlying infection severity, as patients requiring both intraoral and extraoral drainage likely had more extensive abscesses. However, it could also be hypothesized that creating an oral–cutaneous communication could facilitate contamination of the abscess cavity by the oral microbiota, potentially impairing healing and delaying infection resolution. Therefore, when an abscess has already been drained extraorally, additional intraoral drainage requires careful monitoring, given its necessity in specific cases.

This study has some limitations. The first is the retrospective design of the study and its inherent biases, such as information (e.g., unrecorded minor complications) and selection bias. Other factors, such as the relatively small number of patients included and the monocentric nature of the study, should also be considered and may limit generalizability, since institutional protocols and follow-up practices vary. Additionally, potential confounders such as surgeon experience and antibiotic duration were not included in the model due to data limitations, which may influence the results.

## 5. Conclusions

In conclusion, our study reported a 24.1% postoperative complication rate after maxillofacial abscess surgery, which is consistent with previous studies, while the mortality rate (0.4%) was lower than previously reported. Independent risk factors for complications included an ASA score > 2, female sex, and elevated CRP level > 50 mg/L. In high-risk patients, close monitoring during the first five postoperative days is particularly crucial to detect early complications, including persistent abscesses. Additionally, an outpatient follow-up at approximately two weeks after surgery is recommended to identify delayed complications, such as recurrence. However, this follow-up was not standardized in our study and should be validated in future research.

## Figures and Tables

**Table 1:** Description of postoperative complications within 30 days.

Complication	*n* (%)	POD, Median (Range)
Dindo–Clavien grade 1	25 (9.9)	5 (1–16)
Hypokalemia	8 (3.2)	4 (1–12)
Lower-extremity edema	8 (3.2)	4.5 (1–10)
Pleural effusion	2 (0.8)	4.5 (2–7)
Acute pain	2 (0.8)	8.5 (1–16)
Postoperative anemia	1 (0.4)	4
Renal insufficiency	1 (0.4)	5
Herpes angina	1 (0.4)	10
Knee gout	1 (0.4)	14
Urinary retention	1 (0.4)	14
Dindo–Clavien grade 2	18 (7.1)	3 (0–20)
Acute hypertension	5 (2.0)	3 (2–12)
Venous thromboembolism	2 (0.8)	9
Corneal erosion	2 (0.8)	1.5 (1–2)
Delirium	2 (0.8)	12 (4–20)
Drug allergy	2 (0.8)	7 (1–13)
MSSA bacteremia	1 (0.4)	1
Progressive neck swelling	1 (0.4)	8
Postoperative anemia	1 (0.4)	7
NSTEMI	1 (0.4)	2
Tachyarrhythmia	1 (0.4)	0
Dindo–Clavien grade 3a	11 (4.3)	5 (2–15)
Persistent abscess	3 (1.2)	5 (2–5)
Recurrent abscess	3 (1.2)	14 (14–15)
Periodontal abscess	1 (0.4)	6
Intraoral bleeding	1 (0.4)	2
Upper gastrointestinal bleeding	1 (0.4)	6
Epistaxis	1 (0.4)	2
Atrial flutter	1 (0.4)	3
Dindo–Clavien grade 3b	19 (7.5)	9 (2–30)
Persistent abscess	10 (4.0)	4 (2–15)
Recurrent abscess	8 (3.2)	15 (9–30)
Bleeding from the temporal incision	1 (0.4)	10
Dindo–Clavien grade 4a	9 (3.6)	2 (0–5)
Airway compromise due to swelling of the neck	1 (0.4)	0
Persistent collection with airway compromise	2 (0.8)	1 (0–2)
Decompensated heart failure	2 (0.8)	3.5 (3–4)
Blood aspiration following an epistaxis	1 (0.4)	0
Mediastinitis	1 (0.4)	5
Hospital-acquired pneumonia	1 (0.4)	2
Encephalopathy caused by Parkinson disease	1 (0.4)	5
Dindo–Clavien grade 4b	0	
Dindo–Clavien grade 5 (death)	1 (0.4)	4

POD, postoperative day; MSSA, methicillin-sensitive Staphylococcus aureus; NSTEMI, non-ST segment elevation myocardial infarction.

**Table 2:** Univariable analysis for preoperative predictors of postoperative complications.

Characteristic	Overall Complications		Major Complications	
	OR (95%CI)	*p*-Value	OR (95%CI)	*p*-Value
Age > 60 years	2.62 (1.39–4.93)	**0.003**	1.36 (0.63–2.93)	0.434
Sex, female	1.88 (1.05–3.36)	**0.033**	1.36 (0.69–2.70)	0.378
ASA score > 2	3.76 (1.99–7.11)	**<0.001**	3.76 (1.83–7.72)	**<0.001**
BMI, kg/m^2^	1.04 (0.99–1.09)	0.106	1.02 (0.97–1.08)	0.432
Smoking	0.83 (0.46–1.51)	0.546	0.89 (0.44–1.79)	0.740
Immunosuppression	4.02 (1.30–12.46)	**0.016**	2.60 (0.76–8.92)	0.128
Chronic renal failure	4.85 (1.48–15.89)	**0.009**	2.94 (0.84–10.30)	0.091
Diabetes mellitus	1.78 (0.68–4.70)	0.241	2.60 (0.93–7.24)	0.068
Hypertension	1.73 (0.89–3.38)	0.107	1.00 (0.43–2.32)	0.995
Cardiac insufficiency	4.68 (1.56–14.08)	**0.006**	4.68 (1.53–14.36)	**0.007**
Chronic respiratory insufficiency	2.77 (0.81–9.41)	0.103	2.15 (0.54–8.47)	0.276
Psychiatric disorders	1.30 (0.54–3.12)	0.559	1.22 (0.43–3.43)	0.705
Penicillin allergy	1.82 (0.58–5.64)	0.302	1.54 (0.41–5.79)	0.524
Antibiotic therapy prior to admission	1.04 (0.57–1.90)	0.907	0.84 (0.42–1.68)	0.613
Pain duration > three days	1.77 (0.89–3.51)	0.105	1.55 (0.67–3.58)	0.305
Dysphagia	0.73 (0.40–1.33)	0.302	0.87 (0.43–1.77)	0.708
Dyspnea	1.46 (0.35–6.01)	0.604	1.48 (0.30–7.44)	0.631
Trismus (<30 mm)	0.91 (0.50–1.72)	0.769	1.25 (0.57–2.75)	0.577
Previous intraoral drainage	0.74 (0.41–1.36)	0.335	0.70 (0.34–1.44)	0.334
Previous tooth extraction	0.80 (0.41–1.55)	0.510	0.87 (0.40–1.90)	0.734
Preoperative complication	NA		NA	
Fever (>38.0 °C)	0.83 (0.34–2.03)	0.688	0.74 (0.25–2.25)	0.598
White blood cell count > 10 G/L	1.90 (0.92–3.93)	0.082	1.78 (0.74–4.27)	0.194
C-reactive protein level > 50 mg/L	1.98 (0.93–4.19)	0.075	2.00 (0.79–5.04)	0.141
Multiple space involvement	1.50 (0.50–4.49)	0.473	2.80 (0.91–8.56)	0.072
Imputed tooth - Maxillary incisor/canine - Maxillary premolar/molar - Mandibular incisor/canine *- Mandibular premolar/molar (ref.)*	NA NA 0.29 (0.10–0.86)	**0.026**	NA NA 0.91 (0.20–4.29)	0.914

Boldface indicates a statistically significant difference (*p* < 0.05). OR, odds ratio; CI, confidence interval; ASA, American Society of Anesthesiologists; BMI, body mass index; NA, not available (insufficient data); *ref*, reference.

**Table 3:** Univariable analysis for perioperative predictors of postoperative complications.

**Characteristic**	Overall Complications		Major Complications	
	OR (95%CI)	*p*-Value	OR (95%CI)	*p*-Value
Time to operation > 24 h	1.21 (0.65–2.27)	0.554	0.86 (0.39–1.87)	0.702
Operation duration > 30 min	1.48 (0.82–2.64)	0.191	1.43 (0.72–2.87)	0.308
Surgical access - Submental - Cervical + intraoral - Submental + intraoral - Cervical + submental - Cervical + submental + intraoral - Cervical + temporal + intraoral - Temporal + intraoral *- Cervical (ref.)*	0.82 (0.17–4.07) 1.27 (0.68–2.36) 2.47 (0.39–15.65) 2.23 (0.50–9.98) 1.24 (0.12–12.42) NA NA	0.812 0.457 0.336 0.296 0.857	NA 1.54 (0.75–3.17) 1.63 (0.17–15.62) 0.93 (0.11–8.13) NA NA NA	0.240 0.670 0.950
Presence of pus	1.38 (0.73–2.63)	0.320	1.80 (0.81–4.02)	0.152
Additional surgical procedure - Tooth extraction - Tracheotomy - Intraoral drainage - Other *- No (ref.)*	1.07 (0.56–2.03) NA 1.98 (0.76–5.15) 3.70 (0.71–19.40)	0.847 0.164 0.121	1.31 (0.60–2.86) NA 3.27 (1.16–9.23) 1.49 (0.16–13.66)	0.503 **0.026** 0.723
>two extraoral drains	1.54 (0.69–3.47)	0.293	1.36 (0.52–3.58)	0.529
Positive culture	1.88 (0.74–4.78)	0.183	2.45 (0.71–8.46)	0.155

Boldface indicates a statistically significant difference (*p* < 0.05). OR, odds ratio; CI, confidence interval; NA, not available (insufficient data); *ref*, reference.

**Table 4:** Final multivariable logistic model for predictors of postoperative complications.

Characteristic	OR (95%CI)	*p*-Value
*Overall complications*		
Sex, female	1.97 (1.05–3.70)	0.036
ASA score > 2	3.38 (1.75–6.51)	<0.001
C-reactive protein level > 50 mg/L	2.25 (1.01–4.98)	0.046
*Major complications*		
ASA score > 2	3.76 (1.83–7.72)	<0.001

OR, odds ratio; CI, confidence interval; ASA, American Society of Anesthesiologists.

## Data Availability

The data presented in this study are available upon request from the corresponding author.

## Supplementary Materials

The following supporting information can be downloaded at: https://www.mdpi.com/article/10.3390/jcm14103368/s1, File S1: Variable definitions; Table S1: STROBE checklist; Table S2: Demographic and baseline characteristics of 253 patients undergoing extraoral drainage under general anesthesia; Table S3: Perioperative characteristics of 253 patients undergoing extraoral drainage under general anesthesia; Table S4: Description of postoperative complications within 30 days, expanded table.

## Abbreviations

The following abbreviations are used in this manuscript:

ASA	American Society of Anesthesiologists
CI	Confidence interval
CRP	C-reactive protein
CT	Computed tomography
DC	Dindo–Clavien
ICU	Intensive care unit
MSSA	Methicillin-sensitive Staphylococcus aureus
NA	Not available
NaCl	Sodium chloride
NSTEMI	Non-ST segment elevation myocardial infarction
OR	Odds ratio
POD	Postoperative day
ref.	Reference
SD	Standard deviation
STROBE	Strengthening the Reporting of Observational Studies in Epidemiology
