# Supplementary material for: Incidence and Risk Factors for Postoperative Complications in Patients Undergoing Extraoral Drainage for Maxillofacial Abscess: A Retrospective Cohort Study
Source: J Clin Med. 2025 May 12;14(10):3368. doi: 10.3390/jcm14103368 (PMC12111953; doi:10.3390/jcm14103368)
Supplement: Supplementary file 1 [file jcm-14-03368-s001.zip › jcm-3556243-supplementary.pdf]

Supplementary Materials

Table of contents

File S1. Variable definitions. 3

Table S1. STROBE checklist. 4

Table S2: Demographic and baseline characteristics of 253 patients undergoing extraoral drainage under general anesthesia. 6

Table S3. Perioperative characteristics of 253 patients undergoing extraoral drainage under general anesthesia. 8

Table S4. Description of postoperative complications within 30 days, expanded table 9

File S1. Variable definitions.

Variables measured at admission
Age: binary, ≤ 60 years or > 60 years;
Sex: binary, male or female;
American Society of Anesthesiologists (ASA): binary, ≤ two or > two, as defined by Doyle DJ, Hendrix JM, Garmon EH. American Society of Anesthesiologists Classification. [Updated 2022 Dec 4]. In: StatPearls [Internet]. Treasure Island (FL): StatPearls Publishing; 2022 Jan-. Available from: https://www.ncbi.nlm.nih.gov/books/NBK441940. ASA >2 serves as a proxy for overall comorbidities, offering a comprehensive risk assessment beyond individual conditions;
Body Mass Index (BMI): continuous in kg/m2;
Smoking: defined as current or ex-smoker, binary, yes or no;
Immunosuppression: defined as the presence of immunosuppressive therapy (steroid, chemotherapy) or an underlying medical condition (HIV, solid organ transplant), binary, yes or no;
Chronic renal failure, binary, yes or no;
Diabetes mellitus, binary, yes or no;
Hypertension: binary, yes or no;
Cardiac insufficiency: binary, yes or no;
Chronic respiratory insufficiency: binary, yes or no;
Psychiatric disorders: binary, yes or no;
Penicillin allergy: binary, yes or no;
Antibiotic therapy prior to admission: binary, yes or no;
Pain duration: binary, ≤ three days or > three days;
Dysphagia: binary, yes or no;
Dyspnea: binary, yes or no;
Trismus: defined as a mouth opening < 30 mm, binary, yes or no;
Previous intraoral drainage: defined as an intraoral drainage performed before the extraoral drainage under general anesthesia, binary, yes or no;
Previous tooth extraction: defined as extraction of the imputed tooth before the admission, binary, yes or no;
Preoperative complication: defined as the presence of airway obstruction requiring surgical decompression, mediastinitis, septic shock, necrotizing fasciitis, or cavernous sinus thrombosis before the extraoral drainage under general anesthesia, binary, yes or no;
Fever: defined as the presence of temperature > 38.0°C, binary, yes or no;
White blood cell count: binary, ≤10 G/L or >10 G/L;
C-reactive protein: binary, ≤50 mg/L or >50 G/L mg/L;
Multiple space involvement: defined as the involvement of more than one anatomical infected space, based on the imaging or the clinical presentation in case of the absence of preoperative imaging, binary, yes or no;
Imputed tooth: defined as the tooth imputed for the development of the abscess, based on the clinical presentation and/or imaging, categorical: maxillary incisor or canine, maxillary premolar or molar, mandibular incisor or canine, mandibular premolar or molar.

Variables measured during the perioperative course
Time to operation: defined as the difference between the start of surgical incision and the admission to the department of cranio-maxillofacial and oral surgery, binary, ≤24 hours or >24 hours;
Operation duration: defined as the duration from the surgical incision to the wound dressing, binary, ≤30 minutes or > 30 minutes;
Surgical access: categorized as cervical, submental, cervical and intraoral, submental and intraoral, cervical and submental, cervical and submental and intraoral, cervical and temporal and intraoral, or temporal and intraoral;
Presence of pus: defined as the presence of intraoperative purulent discharge, binary, yes or no;
Additional surgical procedure: categorical: none, tooth extraction, tracheotomy, intraoral drainage, or other;
More than two extraoral drains: defined as more than two drains left in situ at the end of the operation, binary, yes or no;
Positive culture: defined as the identification of a specimen from the intraoperative sampling, binary, yes or no.

Table S1. STROBE checklist.

	Item No	Recommendation	Page No
**Title and abstract**	1	(*a*) Indicate the study's design with a commonly used term in the title or the abstract<br>(*b*) Provide in the abstract an informative and balanced summary of what was done and what was found	1
**Introduction**			
Background/rationale	2	Explain the scientific background and rationale for the investigation being reported	1-2
Objectives	3	State specific objectives, including any prespecified hypotheses	2
**Methods**			
Study design	4	Present key elements of study design early in the paper	2
Setting	5	Describe the setting, locations, and relevant dates, including periods of recruitment, exposure, follow-up, and data collection	2
Participants	6	(*a*) Give the eligibility criteria, and the sources and methods of selection of participants. Describe methods of follow-up<br>(*b*) For matched studies, give matching criteria and number of exposed and unexposed	2-3
Variables	7	Clearly define all outcomes, exposures, predictors, potential confounders, and effect modifiers. Give diagnostic criteria, if applicable	2-3
Data sources/ measurement	8*	For each variable of interest, give sources of data and details of methods of assessment (measurement). Describe comparability of assessment methods if there is more than one group	3
Bias	9	Describe any efforts to address potential sources of bias	3
Study size	10	Explain how the study size was arrived at	NA
Quantitative variables	11	Explain how quantitative variables were handled in the analyses. If applicable, describe which groupings were chosen and why	3
Statistical methods	12	(*a*) Describe all statistical methods, including those used to control for confounding<br>(*b*) Describe any methods used to examine subgroups and interactions<br>(*c*) Explain how missing data were addressed<br>(*d*) If applicable, explain how loss to follow-up was addressed<br>(*e*) Describe any sensitivity analyses	3
**Results**			
Participants	13*	(a) Report numbers of individuals at each stage of study—eg numbers potentially eligible, examined for eligibility, confirmed eligible, included in the study, completing follow-up, and analysed<br>(b) Give reasons for non-participation at each stage<br>(c) Consider use of a flow diagram	3-4
Descriptive data	14*	(a) Give characteristics of study participants (eg demographic, clinical, social) and information on exposures and potential confounders<br>(b) Indicate number of participants with missing data for each variable of interest<br>(c) Summarise follow-up time (eg, average and total amount)	3-4, Tables S2
Outcome data	15*	Report numbers of outcome events or summary measures over time	4-6

Main results	16	(*a*) Give unadjusted estimates and, if applicable, confounder-adjusted estimates and their precision (eg, 95% confidence interval). Make clear which confounders were adjusted for and why they were included<br>(*b*) Report category boundaries when continuous variables were categorized<br>(*c*) If relevant, consider translating estimates of relative risk into absolute risk for a meaningful time period	4-7
Other analyses	17	Report other analyses done—eg analyses of subgroups and interactions, and sensitivity analyses	NA
**Discussion**			
Key results	18	Summarise key results with reference to study objectives	8
Limitations	19	Discuss limitations of the study, taking into account sources of potential bias or imprecision. Discuss both direction and magnitude of any potential bias	10
Interpretation	20	Give a cautious overall interpretation of results considering objectives, limitations, multiplicity of analyses, results from similar studies, and other relevant evidence	8-10
Generalisability	21	Discuss the generalisability (external validity) of the study results	8-10
**Other information**			
Funding	22	Give the source of funding and the role of the funders for the present study and, if applicable, for the original study on which the present article is based	10

Reference: von Elm E, Altman DG, Egger M, Pocock SJ, Gøtzsche PC, Vandenbroucke JP; STROBE Initiative. The Strengthening the Reporting of Observational Studies in Epidemiology (STROBE)statement: guidelines for reporting observational studies.
Lancet. 2007 Oct 20;370(9596):1453-7. PMID: 18064739

Table S2: Demographic and baseline characteristics of 253 patients undergoing extraoral drainage under general anesthesia.

Characteristic	n (%) or mean (±SD)
Age > 60 years	
Yes	59 (23.3)
No	194 (76.7)
Sex	
Male	146 (57.7)
Female	107 (42.3)
ASA score > two	
Yes	57 (22.5)
No	192 (75.9)
Missing values	4 (1.6)
BMI, kg/m^2	25.8 (±5.8)
Missing values	3 (1.2)
Smoking	
Yes	161 (63.6)
No	91 (36.0)
Missing value	1 (0.4)
Immunosuppression	
Yes	13 (5.1)
No	240 (94.9)
Chronic renal failure	
Yes	12 (4.7)
No	241 (95.3)
Diabetes mellitus	
Yes	20 (7.9)
No	233 (92.1)
Hypertension	
Yes	52 (20.6)
No	201 (79.5)
Cardiac insufficiency	
Yes	14 (5.5)
No	239 (94.5)
Chronic respiratory insufficiency	
Yes	11 (4.4)
No	242 (95.7)
Psychiatric disorders	
Yes	28 (11.1)
No	225 (88.9)
Penicillin allergy	
Yes	14 (5.5)
No	239 (94.5)
Antibiotic therapy prior to admission	
Yes	115 (45.5)
No	110 (43.5)
Missing values	28 (11.1)
Pain duration > three days	
Yes	107 (42.3)
No	92 (36.4)
Missing values	54 (21.3)
Dysphagia	
Yes	140 (55.3)
No	91 (36.0)
Missing values	22 (8.7)
Dyspnea	
Yes	9 (3.6)
No	223 (88.1)

Missing values	21 (8.3)
Trismus (< 30 mm)	
Yes	159 (62.9)
No	74 (29.3)
Missing values	20 (7.9)
Previous intraoral drainage	
Yes	100 (39.5)
No	148 (58.5)
Missing values	5 (2.0)
Previous tooth extraction	
Yes	70 (27.7)
No	181 (71.5)
Missing values	2 (0.8)
Preoperative complication	
Yes	1 (0.4)
No	252 (99.6)
Fever (> 38.0°C)	
Yes	33 (13.0)
No	217 (85.8)
Missing values	3 (1.2)
White blood cell count > 10 G/L	
Yes	180 (71.2)
No	67 (26.5)
Missing values	6 (2.4)
White blood cell count, G/L	12.7 (4.5)
Missing values	6 (2.4)
C-reactive protein level > 50 mg/L	
Yes	184 (72.7)
No	63 (24.9)
Missing values	6 (2.4)
C-reactive protein level, mg/L	115 (86)
Missing values	6 (2.4)
Multiple space involvement	
Yes	16 (6.3)
No	236 (93.3)
Missing value	1 (0.4)
Imputed tooth	
Maxillary incisor/canine	0
Maxillary premolar/molar	2 (0.8)
Mandibular incisor/canine	14 (5.5)
Mandibular premolar/molar	228 (90.1)
Missing value	9 (3.6)
Length of stay, days	6.6 (±4.0)
Missing value	0

Notes: Continuous variables are mean (±SD) and discrete variables are n (%). Whenever applicable, missing values are reported as n (%).
Abbreviations: SD, standard deviation; ASA, American Society of Anesthesiologists; BMI, body mass index.

Table S3. Perioperative characteristics of 253 patients undergoing extraoral drainage under general anesthesia.

Characteristic	n (%)
Time to operation > 24 hours	
Yes	71 (28.1)
No	181 (71.5)
Missing values	1 (0.4)
Operation duration > 30 minutes	
Yes	120 (47.4)
No	132 (52.2)
Missing values	1 (0.4)
Surgical access	
Cervical	113 (44.7)
Submental	11 (4.4)
Cervical + intraoral	110 (43.5)
Submental + intraoral	5 (2.0)
Cervical + submental	8 (3.2)
Cervical + submental + intraoral	4 (1.6)
Cervical + temporal + intraoral	1 (0.4)
Temporal + intraoral	1 (0.4)
Presence of pus	
Yes	156 (61.7)
No	83 (32.8)
Missing values	14 (5.5)
Additional surgical procedure	
Yes	125 (49.4)
Tooth extraction	94 (37.2)
Tracheotomy	2 (0.8)
Intraoral drainage	23 (9.1)
Other	6 (2.4)
None	127 (50.2)
Missing values	1 (0.4)
> two extraoral drains	
Yes	33 (13.0)
No	214 (84.6)
Missing values	6 (2.4)
Positive culture	
Yes	191 (75.5)
No	37 (14.6)
Missing values	25 (10.0)

Table S4. Description of postoperative complications within 30 days, expanded table.

Complication	n (%)	Therapy	POD, median (range)
Dindo-Clavien grade 1	25 (9.9)		5 (1–16)
Hypokalemia	8 (3.2)	Electrolytes	4 (1–12)
Lower extremity edema	8 (3.2)	Diuretics	4.5 (1–10)
Pleural effusion	2 (0.8)	Diuretics	4.5 (2–7)
Acute pain	2 (0.8)	Analgesics	8.5 (1–16)
Postoperative anemia	1 (0.4)	Iron supplementation	4
Renal insufficiency	1 (0.4)	Fluid resuscitation	5
Herpes angina	1 (0.4)	Topical analgesics	10
Knee gout	1 (0.4)	Analgesics	14
Urinary retention	1 (0.4)	Urinary catheterization	14
Dindo-Clavien grade 2	18 (7.1)		3 (0–20)
Acute hypertension	5 (2.0)	Antihypertensive drug therapy	3 (2–12)
Venous thromboembolism	2 (0.8)	Anticoagulant therapy	9
Corneal erosion	2 (0.8)	Topical antibiotics and anti-inflammatory drugs	1.5 (1–2)
Delirium	2 (0.8)	Antipsychotics	12 (4–20)
Drug allergy	2 (0.8)	Antihistamines and corticosteroids	7 (1–13)
MSSA bacteremia	1 (0.4)	Intravenous antibiotics	1
Progressive neck swelling without fluid collection	1 (0.4)	Intravenous antibiotics	8
Postoperative anemia	1 (0.4)	Blood transfusion	7
NSTEMI	1 (0.4)	Conservative pharmacological treatment $^{\phi}$	2
Tachyarrhythmia	1 (0.4)	Beta-blocker	0
Dindo-Clavien grade 3a	11 (4.3)		5 (2–15)
Persistent abscess	3 (1.2)	Drainage in local anesthesia	5 (2–5)
Recurrent abscess	3 (1.2)	Drainage in local anesthesia	14 (14–15)
Periodontal abscess	1 (0.4)	Drainage in local anesthesia	6
Intraoral bleeding	1 (0.4)	Hemostasis in local anesthesia	2
Upper gastrointestinal bleeding	1 (0.4)	Gastroscopy under sedation	6
Epistaxis	1 (0.4)	Nasal cauterization	2
Atrial flutter	1 (0.4)	Cardioversion under sedation	3
Dindo-Clavien grade 3b	19 (7.5)		9 (2–30)
Persistent abscess	10 (4.0)	Drainage in general anesthesia*	4 (2–15)
Recurrent abscess	8 (3.2)	Drainage in general anesthesia**	15 (9–30)

Bleeding from the temporal incision	1 (0.4)	Hemostasis in general anesthesia	10
Dindo-Clavien grade 4a	9 (3.6)		2 (0–5)
Airway compromise due to swelling of the neck	1 (0.4)	Postoperative transfer to the ICU	0
Persistent collection with airway compromise	2 (0.8)	Drainage in general anesthesia and transfer to the ICU***	1 (0–2)
Decompensated heart failure	2 (0.8)	Hemodynamic support in the ICU****	3.5 (3–4)
Blood aspiration following an epistaxis during the intubation	1 (0.4)	Toilet bronchoscopy in the ICU	0
Mediastinitis	1 (0.4)	Mediastinal drainage and transfer to the ICU	5
Hospital-acquired pneumonia	1 (0.4)	Intravenous antibiotics and transfer to the IC	2
Encephalopathy caused by Parkinson's disease	1 (0.4)	Transfer to the IC	5
Dindo-Clavien grade 4b	0		
Dindo-Clavien grade 5 (death)	1 (0.4)	Organ support in the ICU	4

Notes: POD is expressed as median with range whenever applicable. $^{\phi}$ Catheterization declined in this patient. * Two patients also had their imputed tooth removed. ** Three patients had additionally the imputed tooth removed, among them two also had a partial mandibulectomy. *** One patient had additionally the imputed tooth removed. **** One patient was transferred to the intermediate care.
Abbreviations: POD, postoperative day; MSSA, methicillin-sensitive Staphylococcus aureus; NSTEMI, non-ST-segment elevation myocardial infarction; ICU, intensive care unit; IC, intermediate care.
